# Electron Beam-Assisted Synthesis of YAG:Ce Ceramics

**DOI:** 10.3390/ma16114102

**Published:** 2023-05-31

**Authors:** Zhakyp T. Karipbayev, Victor M. Lisitsyn, Mikhail G. Golkovski, Zhassulan S. Zhilgildinov, Anatoli I. Popov, Amangeldy M. Zhunusbekov, Elena Polisadova, Aida Tulegenova, Dossymkhan A. Mussakhanov, Gulnur Alpyssova, Sergei Piskunov

**Affiliations:** 1 Department of Technical Physics, L.N. Gumilyov Eurasian National University, Astana 010000, Kazakhstan; karipbayev_zht_1@enu.kz (Z.T.K.); zhilgildinov_zhs@enu.kz (Z.S.Z.); zhunusbekov_am@enu.kz (A.M.Z.); 2 Department of Materials Science, Engineering School, National Research Tomsk Polytechnic University, 30, Lenin Ave., Tomsk 634050, Russia; lisitsyn@tpu.ru (V.M.L.); elp@tpu.ru (E.P.); 3 Budker Institute of Nuclear Physics, Siberian Branch, Russian Academy of Sciences, Novosibirsk 630000, Russia; golkovski@mail.ru; 4 Institute of Solid State Physics, University of Latvia, LV-1063 Riga, Latvia; 5 Department of Solid State and Nonlinear Physics, Al-Farabi Kazakh National University, 71, Al-Farabi Ave., Almaty 050040, Kazakhstan; tulegenova.aida@gmail.com (A.T.); piskunov@lu.lv (S.P.); 6 Department of Radiophysics and Electronics, Karaganda Buketov University, Karaganda 100028, Kazakhstan; gulnur-0909@mail.ru

**Keywords:** synthesis, YAG:Ce ceramics, structure, high-power electron flux, luminescence

## Abstract

In this work, we present the results of the structure and luminescence properties of YAG:Ce (Y_3_Al_5_O_12_ doped with Ce^3+^ ions) ceramic samples. Their synthesis was carried out by sintering samples from the initial oxide powders under the powerful action of a high-energy electron beam with an energy of 1.4 MeV and a power density of 22–25 kW/cm^2^. The measured diffraction patterns of the synthesized ceramics are in good agreement with the standard for YAG. Luminescence characteristics at stationary/time-resolved regimes were studied. It is shown that under the influence of a high-power electron beam on a mixture of powders, it is possible to synthesize YAG:Ce luminescent ceramics with characteristics close to the well-known YAG:Ce phosphor ceramics obtained by traditional methods of solid-state synthesis. Thus, it has been demonstrated that the technology of radiation synthesis of luminescent ceramics is very promising.

## 1. Introduction

Yttrium aluminum garnet Y_3_Al_5_O_12_ doped with Ce^3+^ ions (YAG:Ce) phosphor compounds are the most prevalent for the manufacture of white LEDs [1,2,3,4]. YAG-based materials are also applied as scintillators [5,6], in dosimetry [7,8], as well as in various luminescent devices [1,9,10], where they are used in powders, films, ceramics, single crystals, and composites [11,12,13,14,15,16,17,18,19,20]. Materials and structural elements produced based on YAG are high-tech and highly resistant to various external influences, including mechanical, thermal, chemical, and radiation.

The luminescence characteristics of Ce^3+^ ions in garnet compounds differs depending not only on the garnet composition, but also on the method of their crystallization; therefore, the maxima of the Ce^3+^ emission band can be tuned in a fairly wide range of 505–585 nm. To improve the spectral properties of YAG:Ce phosphors, additional elements are often introduced during synthesis for modification, such as Gd^3+^ or other rare earth elements [15,16,17,18,19,20]. 

The synthesis of YAG:Ce material of any morphology is difficult, since the formation of the main structure of yttrium aluminum garnet occurs at temperatures above 1700 °C. Therefore, existing technologies need to be constantly improved, and a constant search for new developments is carried out. Among many proposals, one of the most promising seems to be the use of high-energy particle radiation to accelerate reactions between the initial substance elements of the starting materials to obtain new and more complex materials [21,22,23]. Thus, as usual, leading laboratories and companies are not only developing new production methods, but also improving existing ones.

Of the existing synthesis methods, the most common is the solid-phase synthesis method; this method is currently considered the most optimal and is the most widely used [24,25,26]. Synthesis is carried out from substances with a melting point of more than 2000 °C. From a technological point of view, in order to avoid evaporation of the low-melting compounds, sintering of the initial powders into ceramics is carried out at temperatures of 1500–1700 °C, and only then is long-term annealing repeated at temperatures of about 1700 °C to obtain the predominant YAG phase. However, it should be noted that the reproducibility of the synthesis results is often unsatisfactory. This is because it is difficult to ensure uniform mixing of submicron precursor particles; it is also difficult to grind ceramics to obtain phosphor particles of the same size. Therefore, the improvement of synthesis methods is still not completed and is ongoing.

Among the promising methods being developed, the so-called sol-gel method [27] is well known, and is often used to obtain powders with the required grain sizes. As has been repeatedly confirmed, the sol-gel synthesis produces powders of fine fractions. However, the main disadvantage of this method is that, in addition to the main phase of yttrium-aluminum garnet, the composition also includes some other residual phases of synthesis reactions. Furthermore, the remains of the initial and intermediate synthesis products remain in the phosphor.

Among others, methods of self-igniting synthesis [28] seem very attractive, since the synthesis process in this case takes little time. The “roasting” method is interesting for obtaining powders with submicron particle sizes. However, the disadvantages of these methods are that such combustion processes are poorly controlled; during combustion, additional products are formed from the compositions introduced for combustion, and strong agglomerates of particles are also formed.

Chemical precipitation methods involve the co-precipitation of ceramic components from solution as insoluble salts [29]. The disadvantages of the co-precipitation method are the difficulty of purifying phosphors from residual and intermediate products used in the synthesis; as such, there is clear need for an additional stage of high-temperature annealing, which is necessary to obtain oxides.

Laser sintering has recently been proposed as a new type of heat treatment and molding technology [30]. Laser sintering is capable of not only obtaining complex and precise 3D parts, but also makes it possible to achieve compactness due to high-temperature reactions in a much shorter time, i.e., an order of magnitude smaller than traditional processes.

After preparing the granules, sintering is carried out with a CO_2_ laser with varying power, from 15 to 40 W. Then, they are sintered in a conventional oven at 1500 °C for 12 and 24 h in air.

Among other state-of-the-art methods of transparent ceramic fabrication, it is important to mention the so-called arc plasma melting method [31]. In order to achieve high color rendering lighting, a three-layered phosphor structure was conceived and implemented via the spin coating of BaSi_2_N_2_O_2_:Eu (cyan-emitting) and (Sr,Ca)AlSiN_3_:Eu (red-emitting) phosphor films on the yellow-emitting Y_3_Al_5_O_12_:Ce phosphor ceramic synthesized by the solid-state reaction under vacuum sintering [32]. Lastly, in [33], pore-free YAG:Ce ceramics were obtained through 1700 °C pre-sintering under an N_2_/H_2_ (5%) atmosphere followed by hot isostatic pressing at 1650 °C. The importance of a thorough analysis of porosity and understanding of their evolution has recently been studied in detail by Klym [34].

The above brief review of the main proposed methods for the synthesis of YAG:Ce phosphors shows that the currently existing methods are complex, difficult to control, and also require high-temperature treatment. Various auxiliary materials must be used to facilitate the conversion of the starting raw materials into the phosphor. In addition, these materials and the excess products obtained must be technologically removed from the final product. It is important to note that most of the synthesis methods used so far have been implemented only in laboratory conditions; their industrial promotion is currently not implemented or is not possible for technical reasons.

Recently, the possibility of YAG:Ce ceramics synthesis under powerful influence of high-energy electron beam was successfully demonstrated [31,35]. Radiation synthesis of these ceramics was realized directly from a powder mixture of stoichiometric composition in a time not exceeding 1 s, without using any other substances to facilitate synthesis. Nevertheless, the nature of the processes providing such high synthesis efficiency is not yet fully understood. One of the reasons is that there is not enough information about the structure and properties of the obtained ceramics. In this work, we present the results of the structure and luminescence properties of the YAG:Ce ceramic samples obtained by radiation synthesis in order to compare them with the properties of the samples obtained by traditional methods.

## 2. Materials and Methods

Ceramic samples of different compositions were synthesized with a powder mixture content (Table 1).

**Figure 1:**
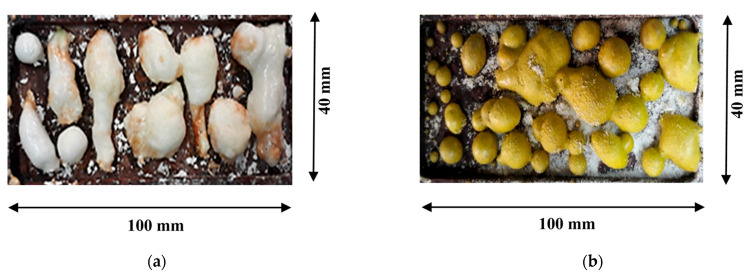
Typical view synthesized of YAG (**a**) and YAG: Ce (**b**) ceramic samples.

The synthesis of ceramics studied in this work was carried out by sintering samples from powders of initial oxides by using the action of a powerful electron beam on them. For such a process, a powerful beam of fast electrons from the ELV-6 accelerator was directed towards a massive copper crucible containing a powder mixture; this apparatus was brought out of a vacuum into a medium with atmospheric pressure through a differential vacuum pumping system. Thus, an electron beam with an energy of 1.4 MeV and a power density of 22–25 kW/cm^2^ performed programmable scanning along the crucible with the speed of 1 cm/s. An electron beam with a cross-section at the crucible surface of 1 cm^2^ converted the crucible into a ceramic sample within 1 s. The crucible was cooled after a single irradiation of the entire surface and the samples were removed from the crucible. Typical pictures of the synthesized YAG (undoped) and YAG:Ce ceramic samples in a 100 × 40 mm crucible are shown in Figure 1. The structure of the crystal lattice of YAG: Ce and YAG:Ce,Gd synthesized ceramics was studied using a Rigaku Miniflex 600 X-ray diffractometer. Reflection spectra were measured on a Jasco 660 spectrophotometer with an integrating sphere. The ceramic samples’ photoluminescence (PL) and luminescence excitation spectra measurements were performed using a Cary Eclipse fluorescence spectrophotometer, Agilent Technologies Inc. (Santa Clara, CA, USA), and a Solar CM2203. 

The PL decay time kinetics were measured using an Andor iSTAR DH734-18F-A3 monochromator and a Tektronix TDS 684A digital oscilloscope. A tunable Ekspla NT 342/3UV laser (5-ns pulse duration and 220–1100 nm spectral range) and a Solar LQ215 + LP603 + LG350 (10-ns pulse duration and 210–2500 nm spectral range) were used for PL excitation. All spectra were measured at RT. Although SEM measurements are not included in this article, relevant measurements on similar samples have been reported [36].

## 3. Results and Discussion

**Figure 2:**
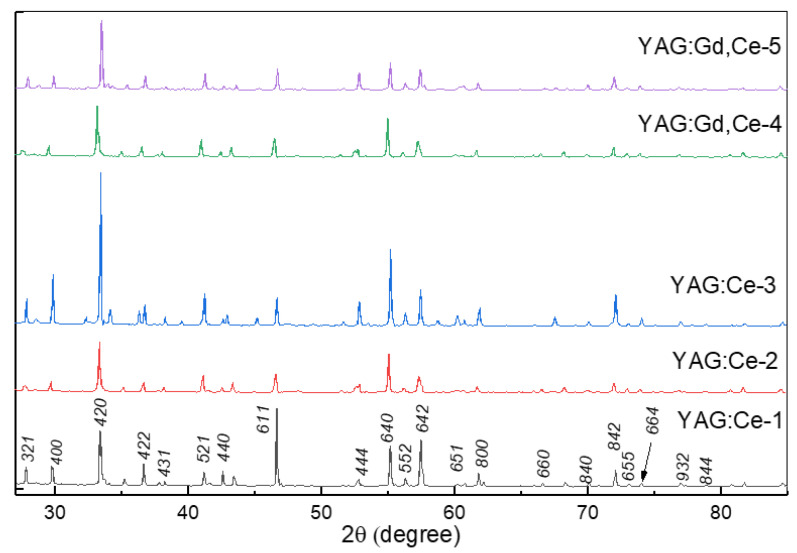
XRD patterns of synthesized YAG: Ce and YAG: Ce,Gd ceramics.

The lattice structure of the YAG: Ce and YAG: Ce synthesized ceramic samples were compared with ICDD (PDF-2 Release 2016 RDB) 01-075-6655. The measured diffraction patterns are in good agreement with known ICDD PDFs. The YAG phase is dominant. Note that a good confirmation of the phase formation is a comparison of measurement results of the lattice parameters in all samples (Table 2, Figure 2). The doping gadolinium as a modifier increases the lattice parameter, which is evidence of its entry into the lattice by replacing yttrium sites. It is clear that the study of the detailed relationship between microstructure and optical properties and morphologies must necessarily be the subject of a special study.

**Figure 3:**
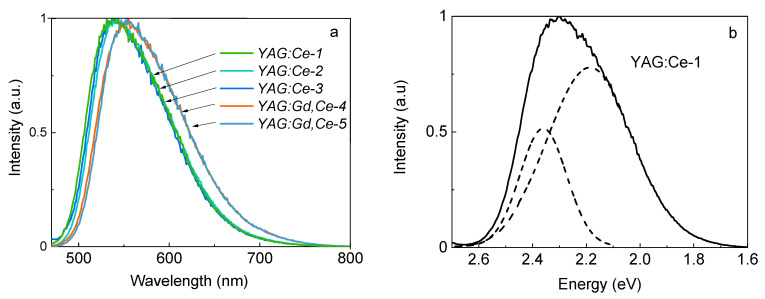
Luminescence spectra excited at 450 nm—(**a**) and Gaussian deconvolution of the luminescence spectrum—(**b**).

We measured the phosphor’s ceramic samples’ excitation and photoluminescence (PL) spectra. The ceramic samples were melted mechanically for research. The resulting powder was poured into washers and lightly pressed so that the surface was even; the powder did not molder during movement. The results of luminescence spectra studies of the samples when excited at 450 nm are shown in Figure 3. The chromaticity diagram is an effective picture for presenting the results. Lighting engineers widely use this picture to characterize light sources and lighting devices, and to calculate lighting installations. However, this diagram does not help to establish the nature of the processes of synthesis and luminescence.

All the studied samples demonstrate rather intense luminescence upon excitation at a wavelength of 450 nm. Similar spectra are also observed upon excitation at a wavelength of 340 nm. The results of the obtained measurements are summarized in Table 3.

Table 3 shows the luminescence spectra measurements results of ceramic samples with the indicated initial mixtures during the synthesis of electron fluxes 22 and 25 kW/cm^2^ with excitation at 450 and 340 nm.

The presented results show that there is an obvious shift in the luminescence spectra band position with gadolinium ion doping as a modifier. It is assumed [37,38,39] that the doping of modifiers leads to a change in the lattice parameter. Accordingly, a change of crystal field in the activator (cerium) ions region leads to its level shifts and the band position to the red region of the spectrum. There are minor changes observed from sample to sample in the band position and their half-widths (see samples 2 and 3). The change in using synthesis flux power leads to a change in the spectrum characteristics within the limits of their spread from sample to sample. This allows us to conclude that a change in the flux power density during synthesis within the used limits does not affect the YAG:Ce phase formation processes. Basically, the luminescence band characteristics are in good agreement with the well-known ones (measured YAG:Ce phosphors and ceramics, obtained by traditional methods [40,41]). 

**Figure 4:**
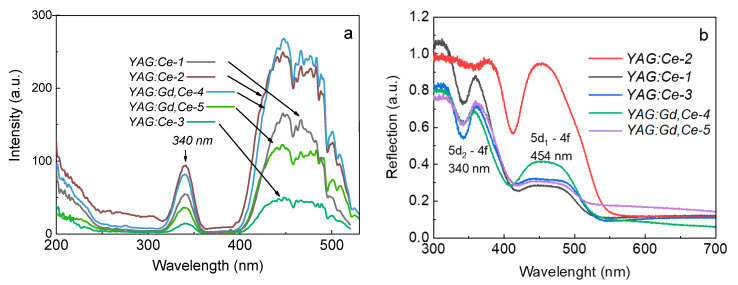
Luminescence excitation spectra of synthesized samples at 540–555 nm (**a**) and reflection/absorption spectra (**b**).

The results of the luminescence excitation spectra of synthesized samples and reflection/absorption spectra are presented in Figure 4. Two bands, at 340 and ~460 nm, caused by ^4^F_5/2_→^5^D_0_, ^5^D_1_ in the Ce^3+^ ions, are observed in the excitation spectra of all samples. Furthermore, excitation by photons at λ ˂ 300 nm initiates the luminescence of Ce^3+^ ions in the region of 500–750 nm. The luminescence intensity increases with decreasing excitation wavelength, i.e., a significant increase in the luminescence intensity is observed as the excitation approaches fundamental absorption. An increase in the excitation intensity in the region from 250 to 200 nm unambiguously indicates the contribution of the Urbach exciton tail, in which the excitons involved in the energy transfer are directly formed. The larger this contribution, the more likely the lower concentration of point defects is in the sample. From the synchrotron reflectivity measurements, it is clearly seen that the Urbach tail contribution begins at 5 eV (or 250 nm) [42,43]. It is interesting to note that optical absorption of the simplest point defects, oxygen vacancies with one or two trapped electrons (the so-called *F^+^* and *F* centers) in all garnets, is also observed in this region of the optical spectrum [44,45,46,47,48].

Thus, through the action of a powerful radiation flux on the powder mixture, it is possible to form YAG:Ce-based luminescent ceramics with characteristics similar to those known YAG:Ce phosphors and ceramics obtained using traditional methods [40,41,49,50,51,52]. 

**Figure 5:**
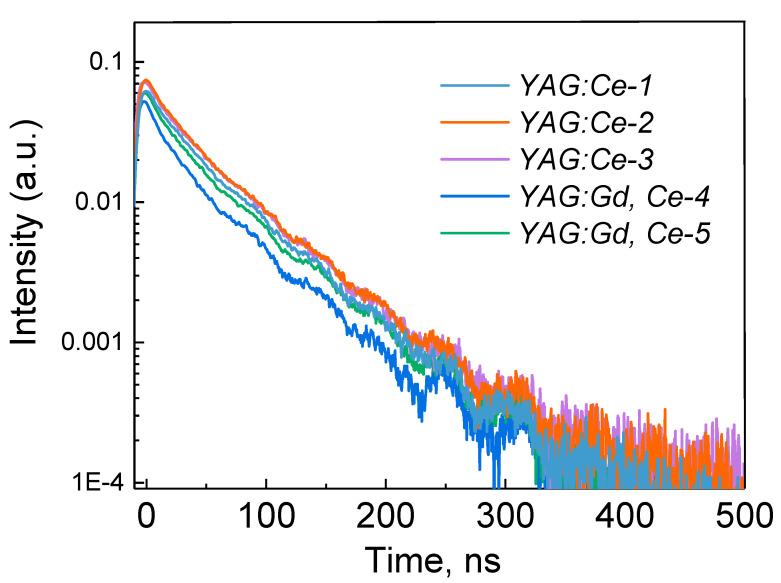
Luminescence excitation spectra of synthesized samples at 540–555 nm.

A nanosecond component is observed in the kinetics of luminescence decay of samples synthesized in a radiation field after pulse excitation at 450 nm (Figure 5, Table 4). The characteristic decay time of the studied samples is within 58–61 ns. The same decay times were also observed upon excitation of the YAG:Ce nanopowders and ceramic samples, which were synthesized by other methods [49,50,53,54,55,56].

As follows from the presented results, the characteristic time of decline of the dominant component of PL depends little on the composition of the sample and the conditions of its synthesis. 

High temperature annealing often improves the optical properties of synthetized ceramic. However, the synthesis of ceramics under electron beam pulse irradiation is fundamentally different from the other methods. High ionization density, and not temperature, is the main factor influencing synthesis under a very intensive electron beam. Synthesis lasts only a second, so the resulting ceramics have an incompletely formed structure. Thus, apparently, heat treatment of the obtained samples is required. It was established in [52] that the luminescence characteristics of the synthetized ceramics depend little on annealing for 8 h at a temperature of 1650 °C. However, annealing changes the color of the ceramic. More information about possible explanation can be found in [52].

## 4. Conclusions

In this way, through the action of a powerful electron beam flux on the powder mixture of several oxides (Al_2_O_3_, Y_2_O_3_, and Ce_2_O_3_), it is possible to form the YAG:Ce-based luminescent ceramics with characteristics similar to those known YAG:Ce phosphors and ceramics obtained by other traditional methods. The formation of ceramics takes place in no more than 1 s, without the use of any other substances to facilitate synthesis. Under the experiment conditions, the synthesis productivity was 0.5 g/s of ceramics. Reasoning about the nature of the processes in radiative synthesis is a very difficult question. We do not have sufficient arguments to prove our existing hypotheses. However, we are already sure that heating during synthesis is not decisive, although heating promotes synthesis. To date, the level of development of the synthesis method is insufficient to establish relationships between the structure and properties. We found that the synthesis is realized in less than 1 s and without the addition of any substances to facilitate the synthesis and only due to the energy of the radiation flux. It was found in [57] that the result of the synthesis depends on the degree of purity, the particle size of the starting material, the flux power density, and the electron energy. Synthesis does not require particles of initial nanosized powders; they will disappear during synthesis due to charging. Synthesis is not realized by heating the mixture, but we do not know how. The radiation synthesis technology makes it possible to obtain samples weighing up to 20–30 g. The technology is now at the initial stage of synthesis development. So far, we can state that the synthesis of luminescent ceramics based on YAG is effectively realized in a radiation field. The resulting activated ceramic luminesces well. In the present work, it is shown that the luminescence characteristics are similar to those known for phosphors obtained by thermal labor-intensive, currently used in the main methods.

The linear high voltage electron accelerator used here has a very high efficiency, exceeding 80%, and its electron flux power can exceed 500 kW [58]. Therefore, radiation technology for the synthesis of dielectric refractory materials can be cheaper. The flaxes of accelerated electrons in magnitude, position, and distribution in three-dimensional coordinate space can be easily controlled using simple devices. Moreover, the change in electron energy can also be easily adjusted. Therefore, it can be said with confidence that the high speed and efficiency of the radiation synthesis of different ceramics and the possibility of controlling of their synthesis process make it possible to consider the radiation synthesis as very promising.

## Figures and Tables

**Table 1:** The composition ratio of the initial oxides is shown in molar %.

Sample	Composition
1. YAG:Ce	Al_2_O_3_ (59.5%) + Y_2_O_3_ (35.7%) + Ce_2_O_3_ (4.8%)
2. YAG:Ce	Al_2_O_3_ (59.5%) + Y_2_O_3_ (35.7%) + Ce_2_O_3_ (4.8%)
3. YAG:Ce	Al_2_O_3_ (59.5%) + Y_2_O_3_ (35.7%) + Ce_2_O_3_ (4.8%)
4. YAG:Ce,Gd	Al_2_O_3_ (59.5%) + Y_2_O_3_ (23.8%) + Gd_2_O_3_ (11.9%) + Ce_2_O_3_ (4.8%)
5. YAG:Ce,Gd	Al_2_O_3_ (59.5%) + Y_2_O_3_ (23.8%) + Gd_2_O_3_ (11.9%) + Ce_2_O_3_ (4.8%)

**Table 2:** Results from XRD analysis of YAG: Ce and YAG: Ce,Gd ceramics.

Sample	Phase	Cell Parameter, Å	Crystallite Size, nm	Crystallinity,%	Phase Content, %
1. YAG:Ce	Y_3_Al_5_O_12_-Cubic Ia-3d(230)	a = 12.01313	46.1	86.3	91.2
	Al_2_O_3_-Rhombo.H.axes-R-3c(167)	a = 4.76400, c = 12.99785	44.4		8.8
2. YAG:Ce	Y_3_Al_5_O_12_-Cubic Ia-3d(230)	a = 11.96366	48.3	91.1	82.4
	Al_2_O_3_-Rhombo.H.axes-R-3c(167)	a = 4.80604, c = 13.04118	44.7		14.9
3. YAG:Ce	Y_3_Al_5_O_12_-Cubic Ia-3d(230)	a = 11.91594	48.5	86.3	100
4. YAG:Ce,Gd	Y_3_Al_5_O_12_-Cubic Ia-3d(230)	a = 11.95920	28.7	88.1	83.0
	Al_2_O_3_-Rhombo.H.axes-R-3c(167)	a = 4.72784, c = 12.93445	42.9		17.0
5. YAG:Ce, Gd	Y_3_Al_5_O_12_-Cubic Ia-3d(230)	a = 12.02285	46.3	86.3	78.5
	Al_2_O_3_-Rhombo.H.axes-R-3c(167)	a = 4.76239, c = 13.02897	44.3		21.5

**Table 3:** The luminescence band characteristics of ceramic samples with excitation at 450 and 340 nm.

Sample #	Electron Flux Power, kW/cm^2^	λ_exc_ = 450 nm		λ _exc_ = 340 nm	
		λ_max_	ΔE, eV	λ_max_	ΔE, eV
1. YAG:Ce	25	539	0.407	538	0.413
2. YAG:Ce	22	542	0.389	542	0.391
3. YAG:Ce	22	538	0.391	538	0.396
4. YAG:Ce,Gd	25	553	0.410	553	0.413
5. YAG:Ce,Gd	22	553	0.395	554	0.393

**Table 4:** The characteristic luminescence decay time after pulsed excitation at 450 nm.

Sample #	Electron Flux Power, kW/cm^2^	τ, ns
1. YAG:Ce	25	59.6
2. YAG:Ce	22	58.0
3. YAG:Ce	22	59.0
4. YAG:Ce, Gd	25	59.0
5. YAG:Ce, Gd	22	60.2

## Data Availability

The data presented in this study are available on request from the corresponding author.
